# Risk prediction of mechanical complications in acute myocardial infarction patients with prior malignancy

**DOI:** 10.3389/fphar.2025.1643770

**Published:** 2025-08-25

**Authors:** Changying Zhao, Chuqing Yang, Yang Yan

**Affiliations:** ^1^ Department of Cardiovascular Surgery, The First Affiliated Hospital of Xi’an Jiaotong University, Xi’an, China; ^2^ Xi’an Jiaotong University Health Science Center, Xi’an, China

**Keywords:** acute myocardial infarction, malignancy, mechanical complications, prediction model, nomogram

## Abstract

**Background:**

Acute myocardial infarction (AMI) patients with prior malignancy have been largely understudied, despite potentially facing higher risks of adverse outcomes. This case-control study aimed to identify independent risk factors for in-hospital mechanical complications among AMI patients with prior malignancies.

**Methods:**

This study enrolled AMI patients with prior malignancy who were hospitalized for treatment. Patients were divided into complication and control groups based on the occurrence of in-hospital mechanical complications. The mechanical complications in this study were defined as papillary muscle rupture (with or without acute mitral regurgitation), ventricular septal defect, large pericardial effusion, left ventricular pseudoaneurysm, and free wall rupture. Relaxed least absolute shrinkage and selection operator (LASSO) logistic regression was used to identify independent risk factors, and Shapley Additive Explanations (SHAP) analysis was employed to evaluate factors. A predictive nomogram was developed based on risk factors and evaluated through internal validation using Bootstrap method with Brier score.

**Results:**

A total of 127 AMI patients with prior malignancy were included, among whom 26 (20.5) were divided in the complication group. The in-hospital mortality was higher in the complication group compared to the control group [2 (7.7%) vs. 0 (0.0%), P = 0.041]. Following baseline analysis, LASSO logistic regression identified six independent risk factors, ranked by SHAP values as follows: body mass index, D-dimer, pulmonary hypertension, wall motion abnormalities, ventricular arrhythmia, and statin use. The nomogram, constructed by these factors, demonstrated good predictive performance, with a Brier score of 0.116 in the internal validation.

**Conclusion:**

This study highlights key clinical predictors for mechanical complications in AMI patients with prior malignancy. The long-term usage of statins might benefit this specific patient population even after the onset of AMI. The proposed nomogram offers a practical tool for early risk assessment and may support improved clinical decision-making.

## 1 Introduction

As a significant global public health issue, cancer presents an exceptionally high disease burden, resulting in approximately 600,000 deaths in the United States in 2020 (Schwartz, 2024). However, studies have found that conventional chemotherapy may adversely impacts the cardiac and vascular systems when effectively suppressing tumor cells. Furthermore, thoracic radiotherapy employed for treating primary or metastatic cancers of the mediastinum and thorax is also associated with various forms of heart disease (Madias, 2023). The treatment of tumors can lead to abnormalities in several cardiovascular markers such as lipid levels, blood pressure, and cardiac enzymes, consequently, patients with tumors are at an elevated risk for developing cardiovascular diseases. Furthermore, advancements in cancer treatment have contributed to a decline in mortality rates, thereby increasing the life expectancy of cancer survivors. A substantial number of individuals with active malignancies or prior malignancy are more likely to experience cardiovascular diseases, which has become another emerging cause of death among cancer patients (Ward et al., 2012).

Among the cardiovascular complications, acute myocardial infarction (AMI) warrants particular attention. The risk of AMI is three times higher in cancer patients compared to those without cancer (Panday et al., 2023). The complex clinical presentations and therapeutic challenges associated with this condition often render single-targeted diagnostic and treatment strategies—whether focused on AMI or malignancy—insufficient to achieve optimal outcomes. This highlights the critical need to carefully balance oncologic therapy with cardiovascular protection, which poses a significant challenge for clinicians who must consider the intricate interplay between these conditions while formulating individualized therapeutic regimens.

Mechanical complications—such as papillary muscle rupture, ventricular septal defects, and free wall rupture—are life-threatening conditions resulting from structural damage to the myocardium due to myocardial necrosis (Damluji et al., 2021). Although the incidence of these complications has significantly declined in the era of coronary reperfusion therapy, they remain associated with high mortality rates (Murphy and Goldberg, 2022). Studies have shown that in the United States, the incidence of mechanical complications following ST-segment elevation myocardial infarction was 0.27%, with an in-hospital mortality rate of 42.4%. For non-ST-segment elevation myocardial infarction, the corresponding data were 0.06% and 18.0%, respectively (Espinoza Alva et al., 2022). Furthermore, both surgical and percutaneous interventions for mechanical complications are highly complex and require coordinated management by a multidisciplinary team. Given the high risk and time-sensitive nature of these conditions, prompt prediction of is crucial to reducing the risk of mortality in this special kind of patients.

Current studies have generally paid limited attention to AMI patients with prior malignancy, who may be at higher risk for adverse outcomes. This study aimed to investigate AMI patients with prior malignancy by stratifying them into complication and control group. The objective was to identify independent risk factors present at admission that could predict the development of mechanical complications during hospitalization.

## 2 Methods

### 2.1 Patient enrollment

This case-control study enrolled AMI patients who had prior malignancy and were hospitalized at the First Affiliated Hospital of Xi’an Jiaotong University between January 2021 and December 2024. The diagnosis of AMI was established in accordance with the criteria outlined in the guidelines from the European Society of Cardiology. The exclusion criteria were as follows: (1) incomplete clinical data that could not confirm the AMI diagnosis; (2) uncertainty regarding the benign or malignant nature of the tumor; (3) missing critical data that could not be supplemented using appropriate statistical imputation methods.

This study was approved by the Ethics Committee of the First Affiliated Hospital of Xi’an Jiaotong University (No. XJTU1AF2025LSYY-606) and was conducted in accordance with the Declaration of Helsinki. The Ethics Committee granted a waiver for informed consent to this study due to the retrospective design.

### 2.2 Data collection and grouping

Basic demographic information (including age and gender), comorbidities (including hypertension, type 2 diabetes mellitus, etc.), biochemical parameters (including blood counts, coagulation-related markers, cardiac enzymes, etc.), electrocardiogram, echocardiographic findings and long-term medications used prior to admission were retrieved from the Biobank of the First Affiliated Hospital of Xi’an Jiaotong University.

Patients were categorized into either the complication group or the control group based on the occurrence of in-hospital mechanical complications. The mechanical complications in this study were defined as follows: papillary muscle rupture (with or without acute mitral regurgitation), ventricular septal defect, large pericardial effusion, left ventricular pseudoaneurysm, and free wall rupture (Damluji et al., 2021; Yoon et al., 2021).

### 2.3 Statistical analysis

Missing data were handled using multiple imputation techniques. Continuous variables that followed a normal distribution were summarized as mean ± standard deviation and compared between groups using Student’s t-test. Variables with non-normal distributions were presented as median and interquartile range (25th–75th percentiles) and analyzed using the Mann–Whitney U test. Categorical variables were reported as absolute frequencies and percentages, and group comparisons were performed using the chi-square test or Fisher’s exact test, as appropriate. Variables with a P value <0.10 in the baseline analysis were entered into a relaxed least absolute shrinkage and selection operator (LASSO) logistic regression model to identify independent risk factors associated with the development of mechanical complications. Considering the limited sample size, 10-fold cross-validation was performed using the lambda.1se criterion to select a more parsimonious model. To enhance the interpretability of the final model and to provide both global and local insights into its predictions, Shapley Additive Explanations (SHAP) analysis was employed. Grounded in cooperative game theory, this approach assigns each feature an importance value for a specific prediction. The SHAP values were calculated based on the final relaxed LASSO model selected through cross-validation. Selected predictors were then used to construct a nomogram for potential clinical application. The discriminative ability of the model was evaluated using the receiver operating characteristic curve. To assess the robustness of the model on the training set, internal validation was performed using the Bootstrap method with 1,000 resamples. The area under the curve (AUC) and its 95% confidence interval (CI) were calculated for both the original and bootstrap-corrected models. Calibration curves were used to evaluate the agreement between predicted probabilities and actual outcomes, showing the calibration performance of both the original and bootstrap-corrected models. Additionally, decision curve analysis was employed to assess the potential clinical utility of the model. The results of the internal validation were evaluated using the Brier score. Statistical analyses were conducted using SPSS software (version 27.0, IBM Corp., Chicago, IL, United States) and R software (version 4.1.1, R Foundation for Statistical Computing, Vienna, Austria). A two-sided P value <0.05 was considered statistically significant.

## 3 Results

### 3.1 Baseline characteristics

A total of 10,210 patients were screened, of whom 127 (1.24%) AMI patients with prior malignancy were finally included. Among these patients, 26 patients developed mechanical complications. The baseline clinical characteristics of the two groups were summarized in Table 1. Compared to the control group, the complication group had a significantly higher proportion of patients with pulmonary hypertension (PH) [6 (23.1%) vs. 2 (2.0%), P < 0.001], as well as a greater prevalence of diuretic use [14 (53.8%) vs. 32 (31.7%), P = 0.036]. Additionally, the complication group showed lower use of angiotensin-converting enzyme inhibitors [3 (11.5%) vs. 35 (34.7%), P = 0.022] and statins [21 (80.8%) vs. 96 (95.0%), P = 0.030] compared to the control group. In terms of laboratory findings, patients in the complication group had significantly lower levels of pro-brain natriuretic peptide (pro-BNP) [2,419.71 (533.58–7,606.75) vs. 7,606.75 (335.70–2,593.50) pg/mL, P = 0.049]. In contrast, D-dimer levels [1.05 (0.65–4.04) vs. 0.71 (0.39–1.26) μg/mL, P = 0.015] were significantly elevated in the complication group. It is worth noting that no significant differences in specific types of malignancy were observed between the complication and control groups, which might be attributed to the limited sample size.

**TABLE 1:** Baseline characteristics and in-hospital outcomes of two groups.

Items	Total (n = 127)	Complication group (n = 26)	Control group (n = 101)	P Value
Age (years)	68.46 ± 9.26	66.96 ± 9.82	68.85 ± 9.12	0.356
Gender (male, %)	100 (78.70)	18 (69.20)	82 (81.20)	0.184
BMI (kg/m^2^)	24.51 ± 3.63	21.93 ± 3.05	25.17 ± 3.48	<0.001
Smoking (%)	45 (35.40)	8 (30.80)	37 (36.60)	0.577
Drinking (%)	22 (17.30)	5 (19.20)	17 (16.80)	0.775
Heart rates (beats/min)	77 (69, 88)	78 (73, 100)	75 (68, 87)	0.107
STEMI (%)	11 (8.70)	4 (15.40)	7 (6.90)	0.234
Killip Ⅲ/Ⅳ (%)	76 (59.80)	15 (57.70)	61 (60.40)	0.802
Types of malignancy (%)				0.721
Lung	32 (25.2)	10 (38.5)	22 (21.8)	
Digestive tract	34 (26.8)	6 (23.1)	28 (27.7)	
Digestive gland	8 (6.3)	2 (7.7)	6 (5.9)	
Urinary system	21 (16.5)	3 (11.5)	18 (17.8)	
Neck	12 (9.4)	1 (3.8)	11 (10.9)	
Female reproductive system	6 (4.7)	1 (3.8)	5 (5.0)	
Breast	13 (10.2)	3 (11.5)	10 (9.9)	
Thymus gland	1 (0.8)	0 (0)	1 (1.0)	
Comorbidities (%)				
Hypertension	76 (59.80)	15 (57.70)	61 (60.40)	0.802
T2DM	35 (27.60)	6 (23.10)	29 (28.70)	0.556
Hyperlipidemia	9 (7.10)	2 (7.70)	7 (6.90)	>0.999
CKD	5 (3.90)	0 (0)	5 (5.00)	0.582
Echocardiology (%)				
LVEF	49.02 ± 13.58	44.65 ± 13.30	50.14 ± 13.49	0.066
Wall motion abnormalities	77 (60.6)	20 (76.9)	57 (56.4)	0.057
PH	8 (6.30)	6 (23.10)	2 (2.00)	<0.001
ECG (%)				
Ventricular arrhythmia	5 (3.90)	3 (11.50)	2 (2.00)	0.058
Atrioventricular block	11 (8.70)	3 (11.50)	8 (7.90)	0.695
Biochemical results				
Glucose (mmol/L)	6.87 (5.53, 8.77)	6.98 (5.12, 10.45)	6.82 (5.57, 8.77)	0.876
HbA1c (%)	6.00 (5.50, 6.80)	5.95 (5.45, 8.03)	6.10 (5.50, 6.80)	0.629
Hb (g/L)	129.18 (118.00, 142.18)	121.23 (108.50, 140.25)	132.00 (119.50, 143.50)	0.094
WBC (10^9/L)	7.56 (5.83, 10.12)	6.42 (4.93, 9.78)	7.83 (6.22, 10.19)	0.160
NEU (10^9/L)	5.56 (3.99, 7.91)	4.36 (3.56, 7.40)	5.72 (4.32, 8.08)	0.130
NEU% (%)	73.76 ± 10.99	72.66 ± 12.05	74.05 ± 10.74	0.567
LYMPH (10^9/L)	1.22 (0.99, 1.62)	1.19 (0.80, 1.66)	1.26 (1.00, 1.59)	0.558
LYMPH% (%)	16.49 (11.31, 23.85)	16.23 (11.78, 27.55)	16.49 (10.74, 23.85)	0.937
NLR	4.57 (2.87, 7.14)	4.64 (2.23, 6.98)	4.57 (2.87, 7.78)	0.849
CRP (mg/L)	18.50 (10.00, 33.60)	21.29 (10.00, 43.25)	17.24 (10.00, 30.57)	0.200
hs-CRP (mg/L)	0.50 (0.09, 2.03)	0.44 (0.11, 1.86)	0.54 (0.09, 2.04)	0.805
PCT (ng/mL)	0.11 (0.05, 0.36)	0.18 (0.07, 0.34)	0.10 (0.05, 0.38)	0.505
AST (U/L)	38.00 (24.00, 82.00)	33.00 (21.00, 76.75)	43.00 (24.50, 96.50)	0.344
ALT (U/L)	27.00 (19.92, 42.00)	25.00 (17.50, 36.89)	27.00 (20.50, 42.50)	0.455
Total protein (g/L)	63.26 ± 7.16	62.45 ± 6.66	63.45 ± 7.30	0.557
Albumin (g/L)	37.04 ± 5.73	36.45 ± 5.56	37.18 ± 6.49	0.589
Globulin (g/L)	26.22 ± 4.29	26.00 ± 6.03	26.27 ± 3.79	0.845
A/G	1.42 (1.24, 1.61)	1.47 (1.13, 1.64)	1.41 (1.27, 1.61)	0.988
eGFR (mL/min/1.73m^2^	89.26 (72.22, 96.36)	80.74 (57.89, 91.34)	90.08 (76.59, 97.50)	0.141
Cr (umol/L)	67.00 (54.00, 86.00)	69.00 (53.25, 96.75)	67.00 (48.00, 83.00)	0.817
BUN (mmol/L)	6.05 (4.57, 7.33)	6.43 (5.01, 7.27)	5.61 (4.56, 7.38)	0.533
LDL (mmol/L)	2.36 ± 0.90	2.56 ± 0.98	2.31 ± 0.88	0.216
HDL (mmol/L)	0.95 ± 0.24	0.96 ± 0.24	0.94 ± 0.22	0.706
TG (mmol/L)	1.18 (0.78, 1.88)	1.07 (0.79, 1.64)	1.21 (0.77, 2.10)	0.590
TC (mmol/L)	4.17 ± 1.17	4.12 ± 1.17	4.36 ± 1.20	0.145
Pro-BNP (pg/mL)	1,644.00 (336.00, 3,556.00)	2,419.71 (533.58, 7,606.75)	7,606.75 (335.70, 2,593.50)	0.049
hs-cTnT (ng/dL)	0.50 (0.09, 2.03)	0.44 (0.11, 1.86)	0.54 (0.09, 2.04)	0.630
CK-MB (U/L)	26.00 (14.00, 91.20)	24.95 (13.78, 57.25)	32.00 (13.80, 91.46)	0.626
CK (U/L)	252.00 (82.00, 778.00)	184.00 (69.75, 638.74)	289.00 (87.50, 798.50)	0.283
LDH (U/L)	271.00 (218.00, 409.00)	270.00 (209.75, 330.50)	278.00 (218.00, 413.00)	0.950
APTT(s)	31.50 (27.20, 36.60)	31.13 (25.80, 34.60)	31.50 (28.00, 36.85)	0.642
PT(s)	13.10 (12.10, 13.80)	12.70 (11.65, 13.83)	13.20 (12.25, 13.80)	0.358
INR	1.02 (0.97, 1.07)	1.00 (0.95, 1.13)	1.02 (0.97, 1.07)	0.682
Fibrinogen (g/L)	3.67 (2.92, 4.42)	3.89 (2.90, 4.28)	3.66 (2.93, 4.43)	0.835
D-dimer (mg/L)	0.76 (0.40, 1.46)	1.05 (0.65, 4.04)	0.71 (0.39, 1.26)	0.015
Treatment				
PCI (%)	26 (20.50)	12 (15.80)	14 (27.50)	0.112
Aspirin (%)	112 (88.20)	21 (80.80)	91 (90.10)	0.189
Clopidogrel (%)	84 (66.10)	17 (65.40)	67 (66.30)	0.927
ACEI (%)	38 (29.90)	3 (11.50)	35 (34.70)	0.022
ARB (%)	38 (29.90)	3 (11.50)	35 (34.70)	0.631
Sacubitril valsartan sodium (%)	27 (21.30)	8 (30.80)	19 (18.80)	0.184
β-blocker (%)	99 (78.00)	21 (80.80)	78 (77.20)	0.698
CCB (%)	10 (7.90)	0 (0)	10 (9.90)	0.212
Diuretics (%)	46 (36.20)	14 (53.80)	32 (31.70)	0.036
Statins (%)	117 (92.10)	21 (80.80)	96 (95.00)	0.030
In-hospital outcomes				
IABP (%)	5 (3.90)	2 (7.70)	3 (3.00)	0.271
Length of hospital stay (days)	4 (3, 7)	4 (3, 8)	5 (3, 7)	0.864
In-hospital mortality (%)	2 (1.60)	2 (7.70)	0 (0)	0.041

BMI, body mass index; STEMI, ST-segment elevation myocardial infarction; T2DM, type 2 diabetes mellitus; CKD, chronic kidney disease; LVEF, left ventricle ejection fraction; PH, pulmonary hypertension; ECG, electrocardiogram; HbA1c, hemoglobin A1c; Hb, hemoglobin; WBC, white blood cell; NEU, neutrophil count; LYMPH, lymphocyte count; NLR, neutrophil-to-lymphocyte ratio; CRP, C-reactive protein; hs-CRP, high-sensitivity C-reactive protein; PCT, procalcitonin; AST, aspartate aminotransferase; ALT: alanine aminotransferase; A/G, albumin/globulin ratio; eGFR, estimated glomerular filtration rate; Cr, creatinine; BUN, blood urea nitrogen; LDL, low-density lipoprotein; HDL, high-density lipoprotein; TG, triglycerides; TC, total cholesterol; Pro-BNP, pro-brain natriuretic peptide; hs-cTnT, high-sensitivity cardiac troponin T; CK-MB, creatine kinase isoenzymes MB; CK, creatine kinase; LDH, lactate dehydrogenase; APTT, activated partial thromboplastin time; PT, prothrombin time; INR, international normalized ratio; FDP, fibrin degradation products; PCI, percutaneous coronary intervention; ACEI, angiotensin-converting enzyme inhibitors; ARB, angiotensin ii receptor blocker; CCB, calcium channel blockers; IABP, intra-aortic ballon pump.

Notably, in-hospital mortality was significantly higher in the complication group compared to the control group [2 (7.7%) vs. 0 (0.0%), P = 0.041]. Among the two fatal cases, one patient showed preoperative echocardiographic evidence of a left ventricular aneurysm and wall motion abnormalities. During coronary angiography, the patient suddenly developed ventricular fibrillation and hypotension. Subsequent echocardiography revealed a large pericardial effusion, suggesting cardiac rupture. The other patient was implanted with an intra-aortic balloon pump after admission and exhibited wall motion abnormalities with an LVEF of 38% on echocardiography. Approximately 1 h after percutaneous coronary intervention, the patient suddenly lost consciousness. Electrocardiography showed electromechanical dissociation, leading to suspicion of free wall rupture.

### 3.2 LASSO-logistic regression and SHAP analysis

**FIGURE 1:**
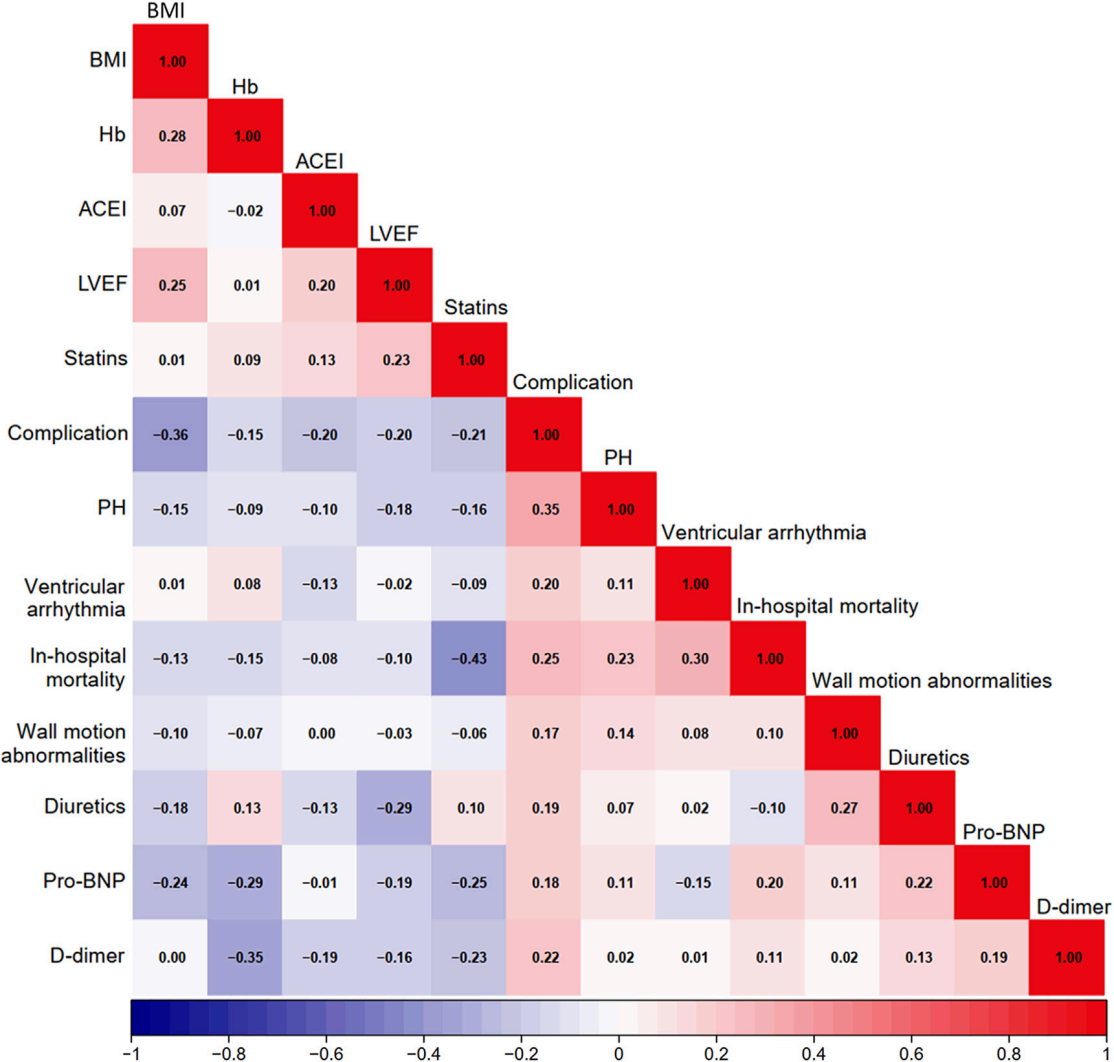
Heatmap of potential factors. BMI, body mass index; Hb, hemoglobin; ACEI, angiotensin-converting enzyme inhibitors; LVEF, left ventricle ejection fraction; PH, pulmonary hypertension; Pro-BNP, pro-brain natriuretic peptide.

**FIGURE 2:**
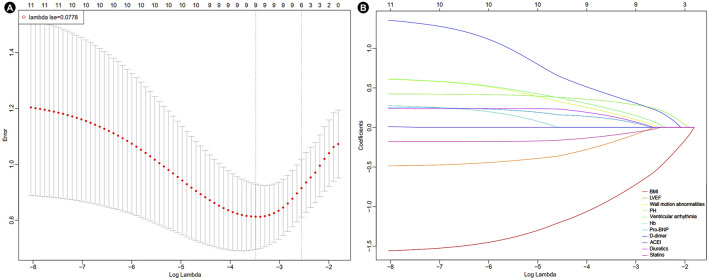
Results of LASSO-logistic regression. **(A)** Cross-validation plot of LASSO-logistic regression. **(B)** Selection process of LASSO-logistic regression model by cross-validation method. BMI, body mass index; LVEF, left ventricle ejection fraction; PH, pulmonary hypertension; Hb, hemoglobin; Pro-BNP, pro-brain natriuretic peptide; ACEI, angiotensin-converting enzyme inhibitors.

A heatmap was used to visualize the correlations among variables that showed a *P* value <0.010 in the baseline analysis (Figure 1). As in-hospital mortality typically occurred following the development of mechanical complications, it was excluded from the following selection. Other variables were further evaluated using LASSO logistic regression. At lambda.1se = 0.0778, six independent risk factors were identified: body mass index (BMI), wall motion abnormalities, PH, ventricular arrhythmia, D-dimer level, and usage of statins (Figure 2; Table 2).

**TABLE 2:** Results pf LASSO-logistic regression model.

Items	LASSO-logistic regression	
	Assignment	Coefficient
BMI	Continuous variable	−1.4428
LVEF	Continuous variable	
Wall motion abnormalities	Yes = 1, No = 0	0.7056
PH	Yes = 1, No = 0	0.5342
Ventricular arrhythmia	Yes = 1, No = 0	0.5081
Hb	Continuous variable	
Pro-BNP	Continuous variable	
D-dimer	Continuous variable	1.3889
ACEI	Use = 1, No = 0	
Diuretics	Use = 1, No = 0	
Statins	Use = 1, No = 0	−0.2446

LASSO, least absolute shrinkage and selection operator; other abbreviations as in Table 1.

**FIGURE 3:**
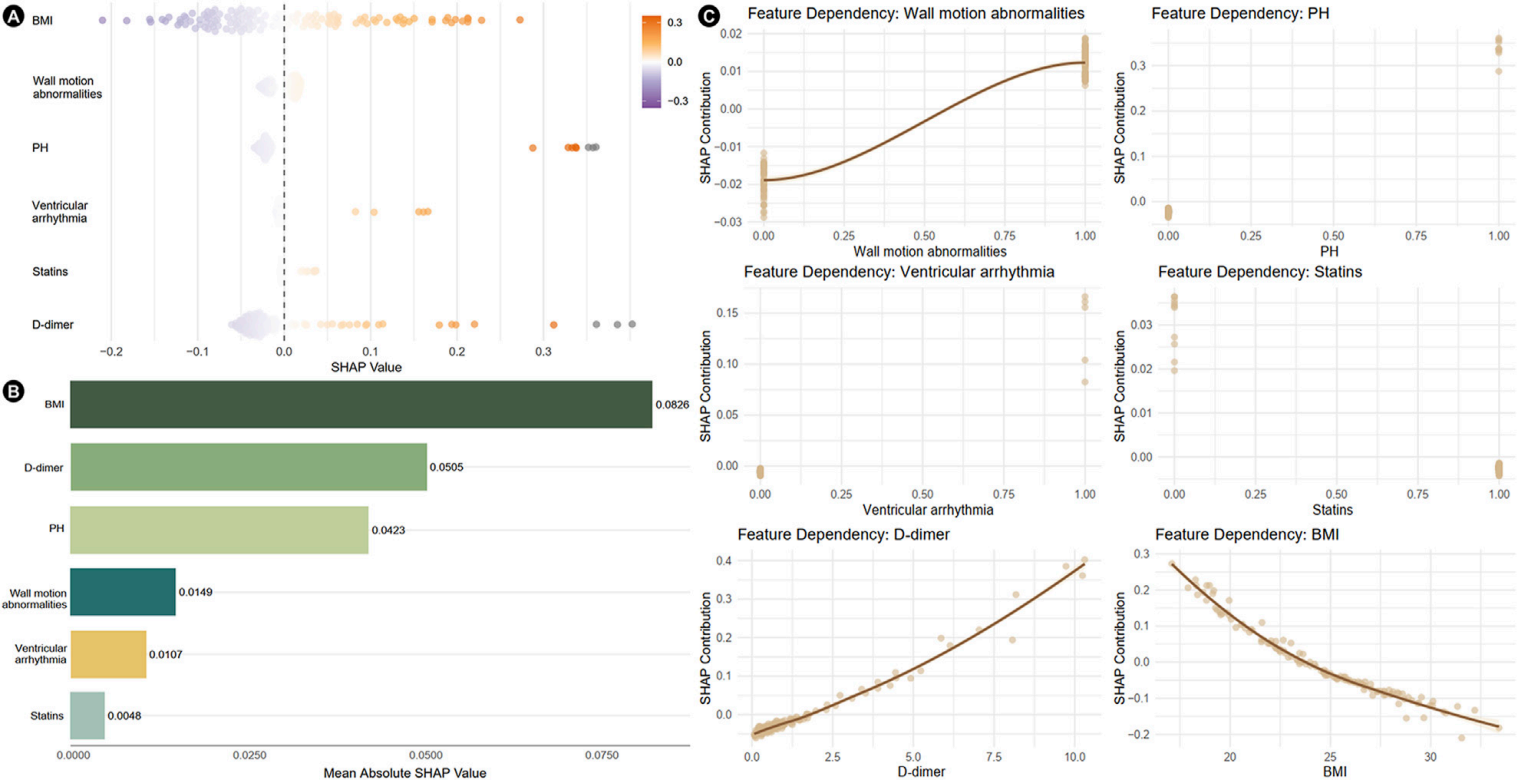
Results of SHAP analysis of independent risk factors. **(A)** Beeswarm plots showing the distribution of individual feature impacts. **(B)** Bar plot ranked by mean absolute SHAP values, illustrating global feature importance. **(C)** Feature dependency plots depicting the relationship between feature values and SHAP values. SHAP, shapley additive explanations; PH, pulmonary hypertension; BMI, body mass index.

The SHAP analysis revealed feature-specific risk patterns (Figure 3). The beeswarm plot demonstrated individualized predictor effects and interaction dynamics (Figure 3A). BMI (mean |SHAP| = 0.0826) emerged as the most influential determinant, followed by D-dimer levels (mean |SHAP| = 0.0505), pulmonary hypertension (PH, mean |SHAP| = 0.0423), wall motion abnormalities (mean |SHAP| = 0.0149), and ventricular arrhythmia (mean |SHAP| = 0.0107). Statin use demonstrated minimal protective effects (mean |SHAP| = 0.0048) (Figure 3B). The feature dependency plots further quantified the nonlinear relationships between key predictors and model outcomes, with SHAP contributions uncovering clinically significant thresholds (Figure 3C).

### 3.3 Construction of nomogram

**FIGURE 4:**
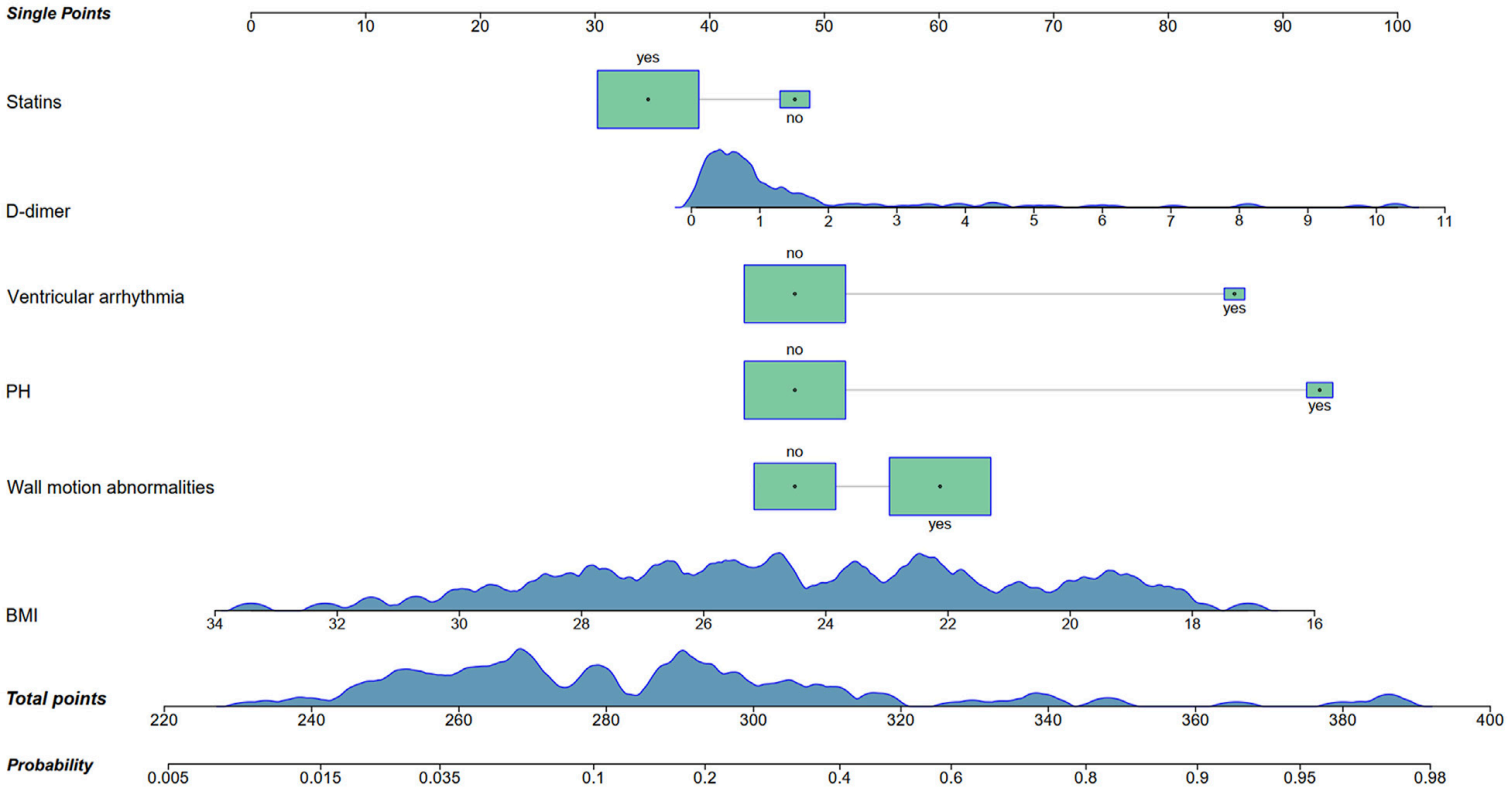
Nomogram for the prediction of mechanical complications in acute myocardial infarction patients with prior malignancy. PH, pulmonary hypertension; BMI, body mass index.

A nomogram was developed based on the six identified variables to predict the likelihood of mechanical complications occurring during hospitalization, using baseline characteristics at admission (Figure 4). Each of the six variables was assigned a corresponding single score according to its clinical and biochemical status. By summing all the single scores, a total score was obtained, which corresponded to the predicted probability of mechanical complications.

**FIGURE 5:**
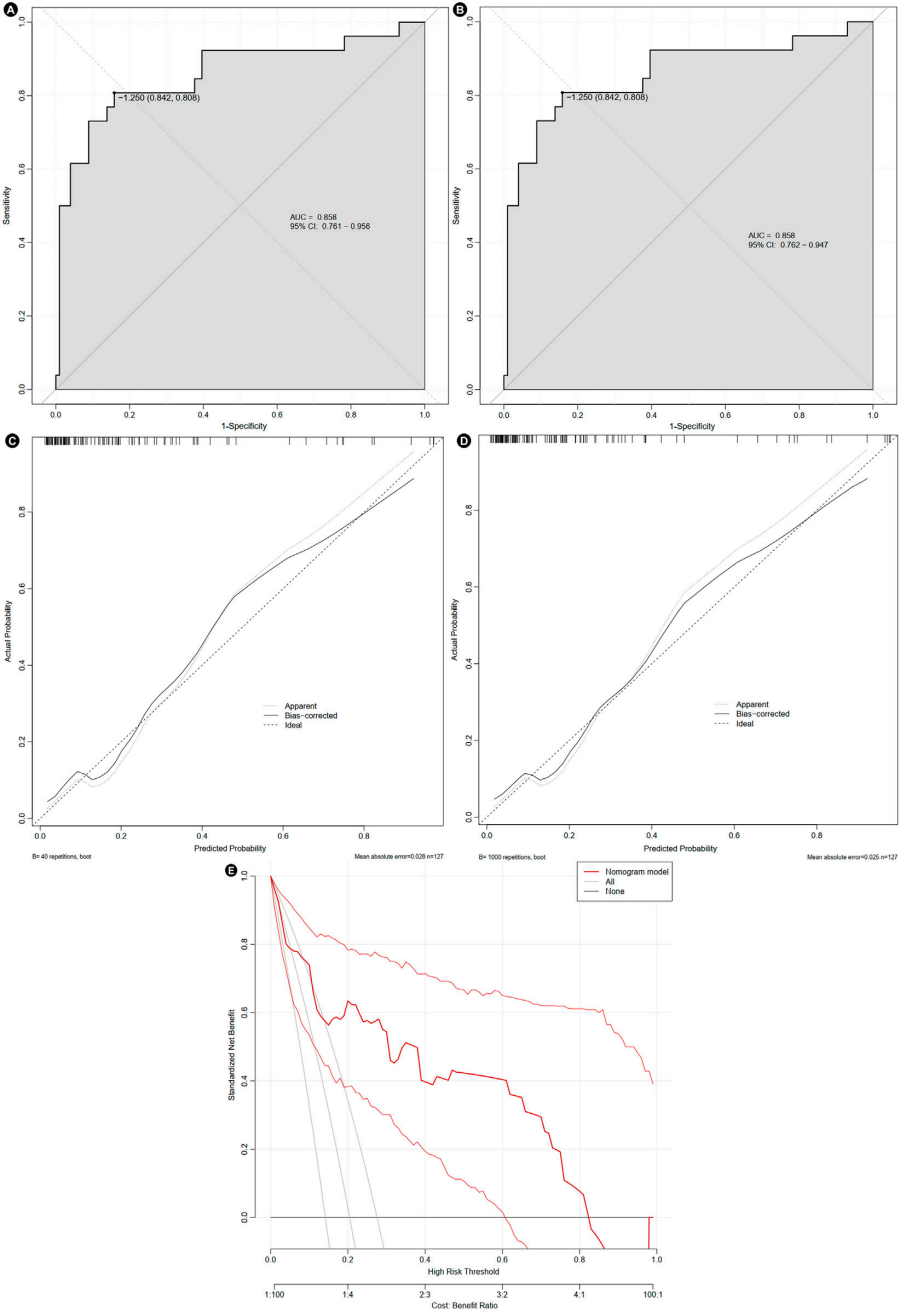
Evaluation of the nomogram. The receiver operating characteristic curve before **(A)** and after bootstrap **(B)**, calibration curve before **(C)** and after bootstrap **(D)**, and decision curve **(E)**. AUC, area under curve; CI, confidence interval.

The area under the AUC for the nomogram prediction model was 0.858 (95% CI: 0.761–0.956). After internal validation using Bootstrap resampling, the AUC was 0.858 (95% CI: 0.762–0.947). These results indicated that the predictive performance of nomogram remained stable even under resampling (Figures 5A,B). The calibration curve indicated that the model had good predictive consistency. After bootstrap internal validation, the model remained well-calibrated, suggesting that its predicted probabilities were stable and reliable, with no evidence of significant overfitting (Figures 5C,D). Bootstrap internal validation resulted in a Brier score of 0.116, indicating satisfactory model calibration and close alignment between predicted probabilities and observed rates. However, the relatively small sample size might have affected the stability of the decision curve analysis. The clinical net benefit estimates across different threshold probabilities showed some variability (Figure 5E).

## 4 Discussion

This study included AMI patients with prior malignancy and divided them into two groups based on the occurrence of mechanical complications. A total of six independent risk factors were finally identified, and a nomogram was constructed to predict the risk of mechanical complications. The findings indicated that statins might reduce the risk of mechanical complications after the onset of AMI in patients with prior malignancy, providing additional benefits for patients.

AMI patients with prior malignancy represent a distinct and often overlooked clinical population. Emerging evidence suggests that the elevated risk of cardiovascular diseases in cancer patients may be driven by chronic systemic inflammation. It is well established that individuals with malignancies frequently exist in a persistent pro-inflammatory state, which can contribute to the initiation and progression of cardiovascular complications. This inflammatory milieu may promote the formation and destabilization of atherosclerotic plaques, leading to the subsequent release of elevated levels of pro-inflammatory cytokines, including interferon-γ, interleukin-1β, interleukin-6, and tumor necrosis factor-α. These cytokines activate multiple intracellular signaling pathways, such as adenosine 5′-monophosphate-activated protein kinase, Janus kinase, and protein kinase B. The activation of these pathways results in the transcriptional upregulation of reactive oxygen species and proteolytic enzymes, which can induce DNA damage and genomic instability (Prousi et al., 2023). Through modulation of key signaling nodes, this cascade may further influence the expression of oncogenes and tumor suppressor genes, thereby linking chronic inflammation not only to cancer progression but also to increased cardiovascular vulnerability. In addition, evidence have suggested that cardiovascular diseases may also influence the tumor development and progression. An animal experimental study revealed that heart failure resulting from AMI can promote the growth of intestinal tumors in mice, which may be associated with some secreted cardiac proteins—particularly SerpinA3. Subsequent *in vivo* studies in mice also demonstrated that cardiac remodeling following AMI may serve as an acute pathological stressor capable of inducing the growth of breast and lung cancers. The underlying mechanism involves the induction of an immune-suppressive state caused by AMI (Fontvieille et al., 2023). Understanding these shared pathophysiological mechanisms is crucial for improving risk stratification and therapeutic strategies in this high-risk patient cohort.

Lipid metabolism also plays a critical role in the progression of cardiovascular disease among cancer patients (Liu et al., 2023). Cancer cells often exhibit profound alterations in lipid metabolic pathways, characterized by the accumulation of elongated and saturated fatty acid chains, which contribute to resistance against apoptosis. Moreover, enhanced isoprenoid production, catalyzed by acetyl-coenzyme A in tumor cells, promotes cholesterol biosynthesis—an effect that may further predispose to atherosclerosis and AMI. Notably, AMI and malignancies share several key pathogenic mechanisms, including dysregulation of reactive oxygen species, activation of the Wnt signaling pathway, and upregulation of hypoxia-inducible factor-1α. These overlapping molecular pathways highlight the complex interplay between cardiovascular and oncologic diseases (Rocca et al., 2023). Mitochondrial dysfunction may contribute to the increased susceptibility of cancer patients to cardiovascular diseases. Patients with both cancer and cardiovascular diseases often show impaired mitochondrial function and dynamics, leading to dysregulation of bioenergetics, metabolism, and intracellular signaling pathways involving reactive oxygen species and Ca^2+^. Moreover, several anticancer therapies can induce mitochondrial stress or dysfunction, potentially resulting in severe cardiovascular complications (Rocca et al., 2023). In summary, cancer patients are more prone to AMI, resulting in poorer prognoses.

The mechanism by which statins reduce the risk of myocardial infarction is closely associated with their well-documented pleiotropic effects. Beyond their lipid-lowering properties through inhibition of 3-hydroxy-3-methylglutaryl coenzyme A reductase and subsequent reduction in cholesterol synthesis, statins exert a range of non-lipid-related beneficial effects. These include anti-inflammatory actions, attenuation of oxidative stress, improvement of vascular endothelial function, and stabilization of atherosclerotic plaques (Das and Freedland, 2023). The antitumor mechanisms of statins may vary across different cancer types. Currently, these mechanisms are believed to mainly include the following pathways: inducing apoptosis, regulating autophagy, targeting the tumor microenvironment, and inducing ferroptosis (Jiang et al., 2021). One important mechanism involves the modulation of protein isoprenylation—an essential post-translational modification required for the activation of key signaling proteins such as Ras and Rho, which are involved in tumor progression. By inhibiting the mevalonate pathway, statins may also impair the YAP/TAZ-dependent transcriptional response, thereby suppressing cancer cell proliferation, regulating the cell cycle, inducing apoptosis, and inhibiting tumor angiogenesis (Jiang et al., 2021). Furthermore, emerging evidence suggests that the reduction of serum cholesterol may serve as an indirect mechanism linking statin use with a lower risk of advanced and fatal prostate cancer (Craig et al., 2022). Clinical studies have reported similar beneficial effects. For instance, a clinical study found that statin use was associated with improved overall survival in patients with pancreatic ductal adenocarcinoma, particularly among those who underwent surgical resection (Tamburrino et al., 2020). Although statins are generally well tolerated, studies have reported that 5%–30% of patients are intolerant to these medications, which may increase the risk of adverse cardiovascular outcomes (Cheeley et al., 2022). Additionally, another research reported that statin use in male patients might be associated with an increased risk of developing kidney cancer (Zeng et al., 2024). Generally, the findings of this study further expand the clinical relevance of statin therapy by providing novel evidence that statins may play a protective role in preventing mechanical complications in patients with both a history of acute myocardial infarction and prior malignancy.

In addition, PH and coagulation dysfunction were found to significantly increase the risk of mechanical complications in this study. This finding is highly consistent with existing clinical observations reported in the literature. From a pathophysiological perspective, adverse vascular remodeling in the pulmonary arteries may lead to increased vascular resistance, which in turn elevates right ventricular afterload and ultimately contributes to the development of right heart failure (Luna-López et al., 2022). Abnormalities in coagulation function are particularly relevant in patients with both malignancy and AMI, and should not be overlooked. In this study, patients in the complication group exhibited shorter activated partial thromboplastin time and prothrombin time, along with elevated fibrin degradation products, compared to the control group. These findings are partially supported by previous studies. For instance, activation of the coagulation system has been documented in plasma samples from patients with localized and/or advanced breast cancer, suggesting a hypercoagulable state in certain tumor populations (Dirix et al., 2022).

However, this study found that a lower body mass index (BMI) was associated with an increased risk of mechanical complications following AMI—an observation that contradicts conventional findings in the general AMI population, where higher BMI is often linked to worse cardiovascular outcomes. This discrepancy may be attributed to the unique clinical profile of cancer survivors. The treatment of malignancy may result in muscle loss due to a combination of the tumor-induced metabolic alterations, systemic inflammation, and treatment-related gastrointestinal toxicity, that affects nutrient intake and other regulatory functions. Low muscle mass is estimated to affect 25%–60% of patients at the time of cancer diagnosis, with higher prevalence observed in certain malignancies, such as lung and head and neck malignancies (Kiss et al., 2023). Moreover, cancer is widely recognized as a wasting disease that promotes malnutrition, which in turn impairs collagen synthesis and compromises tissue repair capacity (Grada and Phillips, 2022). These above factors may compromise myocardial structural integrity and increase susceptibility to mechanical complications after myocardial infarction. Alternatively, BMI may not adequately reflect fat-free mass or body composition, which are more accurate indicators of functional status and cardiorespiratory reserve (Alebna et al., 2024). Furthermore, several studies have reported the complex influence of obesity status on the clinical outcomes in cancer patients. One study indicated that obesity was significantly associated with a more favorable prognosis in breast cancer patients, and an increased risk of mortality following breast cancer was only observed in women with severe obesity (BMI ≥35 kg/m^2^) (Chlebowski et al., 2024). Therefore, in patients with prior malignancy, traditional anthropometric measures such as BMI may fail to reflect the underlying metabolic and physiological risks. Alternative body composition metrics—such as the fat-to-lean weight ratio and hand-grip strength—may offer more insights. A study has suggested that hand-grip strength can serve as a potential marker for cardiometabolic risk (Gerber et al., 2022). Meanwhile, fat-to-lean weight ratio can assess the balance between adipose and lean body mass, thereby providing a more comprehensive evaluation of cardiometabolic health (Aggarwal et al., 2024). In conclusion, the association between low BMI and higher risk of mechanical complications further highlights the distinct pathophysiological characteristics of AMI patients with a prior malignancy. These findings suggest the need for tailored risk assessment tools and updated clinical management strategies that account for the unique vulnerabilities of this patient population.

This study has some limitations. Firstly, among 10,210 screened AMI patients, only 127 (1.24%) had prior malignancy. Though this study screened a large number of AMI patients, the sample size is relatively small, which might have potential impact on the variable selection, nomogram stability and the risk of overfitting. Additionally, future studies with larger samples might elucidate potential associations between specific malignancies and the risk of mechanical complications. Secondly, due to the retrospective design of this study, some characteristics were unable to obtain. Additionally, external validation should be conducted in future research to further evaluate the performance of the nomogram. More attention is needed to investigate the effect of tumor in the onset of mechanical complication in AMI patients. Future prospective studies are warranted to validate these findings and further refine risk prediction models for broader clinical application.

## 5 Conclusion

In summary, this study demonstrates that AMI patients with prior malignancy are at significant risk for in-hospital mechanical complications, which are associated with higher mortality and clinical complexity. Six independent risk factors—BMI, wall motion abnormalities, PH, ventricular arrhythmia, elevated D-dimer levels, and non-use of statins—were identified upon admission. Statins, which are widely used in cardiovascular disease management for their lipid-lowering and pleiotropic effects, appeared to confer a protective role in AMI patients with prior malignancy. These findings underscore the importance of early recognition and tailored management in this high-risk population. The predictive nomogram developed in this study indicated relatively good discrimination and calibration, offering a practical tool for clinicians to assess individual risk and guide timely interventions.

## Data Availability

The raw data supporting the conclusions of this article will be made available by the authors, without undue reservation.

## References

[B1] Aggarwal P. Kuppusamy S. Prakash P. Subramanian S. Fredrick J. (2024). Is fat-to-lean mass ratio a better predictor of heart variability than body mass index? J. Educ. Health Promot 13, 6. 10.4103/jehp.jehp_539_23 38525216 PMC10959267

[B2] Alebna P. L. Mehta A. Yehya A. daSilva-deAbreu A. Lavie C. J. Carbone S. (2024). Update on obesity, the obesity paradox, and obesity management in heart failure. Prog. Cardiovasc Dis. 82, 34–42. 10.1016/j.pcad.2024.01.003 38199320

[B3] Cheeley M. K. Saseen J. J. Agarwala A. Ravilla S. Ciffone N. Jacobson T. A. (2022). NLA scientific statement on statin intolerance: a new definition and key considerations for ASCVD risk reduction in the statin intolerant patient. J. Clin. Lipidol. 16, 361–375. 10.1016/j.jacl.2022.05.068 35718660

[B4] Chlebowski R. T. Aragaki A. K. Pan K. Simon M. S. Neuhouser M. L. Haque R. (2024). Breast cancer incidence and mortality by metabolic syndrome and obesity: the Women's health initiative. Cancer 130, 3147–3156. 10.1002/cncr.35318 38736319

[B5] Craig E. L. Stopsack K. H. Evergren E. Penn L. Z. Freedland S. J. Hamilton R. J. (2022). Statins and prostate cancer-hype or hope? The epidemiological perspective. Prostate Cancer Prostatic Dis. 25, 641–649. 10.1038/s41391-022-00554-1 35732821 PMC9705231

[B6] Damluji A. A. van Diepen S. Katz J. N. Menon V. Tamis-Holland J. E. Bakitas M. (2021). Mechanical complications of acute myocardial infarction: a scientific statement from the American heart association. Circulation 144, e16–e35. 10.1161/CIR.0000000000000985 34126755 PMC9364424

[B7] Das S. Freedland S. J. (2023). Statins and cancer prevention-association does not mean causation. Cancer Prev. Res. (Phila) 16, 1–3. 10.1158/1940-6207.CAPR-22-0420 36597731 PMC10017018

[B8] Dirix L. Y. Oeyen S. Buys A. Liégois V. Prové A. Van De Mooter T. (2022). Coagulation/fibrinolysis and circulating tumor cells in patients with advanced breast cancer. Breast Cancer Res. Treat. 192, 583–591. 10.1007/s10549-021-06484-1 35132503 PMC8960658

[B9] Espinoza Alva D. Mallma Gómez M. Y. Muñoz Moreno J. M. (2022). Mechanical complications after myocardial infarction in a national Reference Hospital. Arch. Peru. Cardiol. Cir. Cardiovasc 3, 25–32. 10.47487/apcyccv.v3i1.200 37408602 PMC10318997

[B10] Fontvieille E. Viallon V. Recalde M. Cordova R. Jansana A. Peruchet-Noray L. (2023). Body mass index and cancer risk among adults with and without cardiometabolic diseases: evidence from the EPIC and UK biobank prospective cohort studies. BMC Med. 21, 418. 10.1186/s12916-023-03114-z 37993940 PMC10666332

[B11] Gerber M. Ayekoé S. Bonfoh B. Coulibaly J. T. Daouda D. Gba B. C. (2022). Is grip strength linked to body composition and cardiovascular risk markers in primary schoolchildren? cross-Sectional data from three African countries. BMJ Open 12, e052326. 10.1136/bmjopen-2021-052326 35667732 PMC9171173

[B12] Grada A. Phillips T. J. (2022). Nutrition and cutaneous wound healing. Clin. Dermatol 40, 103–113. 10.1016/j.clindermatol.2021.10.002 34844794

[B13] Jiang W. Hu J. W. He X. R. Jin W. L. He X. Y. (2021). Statins: a repurposed drug to fight cancer. J. Exp. Clin. Cancer Res. 40, 241. 10.1186/s13046-021-02041-2 34303383 PMC8306262

[B14] Kiss N. Prado C. M. Daly R. M. Denehy L. Edbrooke L. Baguley B. J. (2023). Low muscle mass, malnutrition, sarcopenia, and associations with survival in adults with cancer in the UK biobank cohort. J. Cachexia Sarcopenia Muscle 14, 1775–1788. 10.1002/jcsm.13256 37212184 PMC10401543

[B15] Liu J. Chen S. Zhou Y. Huang H. Li Q. Liang Y. (2023). Proportion and number of incident cancer deaths in coronary artery disease. Cancer Med. 12, 20140–20149. 10.1002/cam4.6595 37754571 PMC10587929

[B16] Luna-López R. Ruiz Martín A. Escribano Subías P. (2022). Pulmonary arterial hypertension. Med. Clin. Barc. 158, 622–629. 10.1016/j.medcle.2022.05.010 35279313

[B17] Madias J. E. (2023). Right *versus* left breast radiation and coronary artery disease: is there a differential? Acta Cardiol. 78, 5–12. 10.1080/00015385.2022.2141431 36378524

[B18] Murphy A. Goldberg S. (2022). Mechanical complications of myocardial infarction. Am. J. Med. 135, 1401–1409. 10.1016/j.amjmed.2022.08.017 36075485

[B19] Panday P. Hausvater A. Pleasure M. Smilowitz N. R. Reynolds H. R. (2023). Cancer and myocardial infarction in women. Am. J. Cardiol. 194, 27–33. 10.1016/j.amjcard.2023.02.007 36931164 PMC10984272

[B20] Prousi G. S. Joshi A. M. Atti V. Addison D. Brown S. A. Guha A. (2023). Vascular inflammation, cancer, and cardiovascular diseases. Curr. Oncol. Rep. 25, 955–963. 10.1007/s11912-023-01426-0 37261651 PMC12258637

[B21] Rocca C. Soda T. De Francesco E. M. Fiorillo M. Moccia F. Viglietto G. (2023). Mitochondrial dysfunction at the crossroad of cardiovascular diseases and cancer. J. Transl. Med. 21, 635. 10.1186/s12967-023-04498-5 37726810 PMC10507834

[B22] Schwartz S. M. (2024). Epidemiology of cancer. Clin. Chem. 70, 140–149. 10.1093/clinchem/hvad202 38175589

[B23] Tamburrino D. Crippa S. Partelli S. Archibugi L. Arcidiacono P. G. Falconi M. (2020). Statin use improves survival in patients with pancreatic ductal adenocarcinoma: a meta-analysis. Dig. Liver Dis. 52, 392–399. 10.1016/j.dld.2020.01.008 32113888

[B24] Ward K. K. Shah N. R. Saenz C. C. McHale M. T. Alvarez E. A. Plaxe S. C. (2012). Cardiovascular disease is the leading cause of death among endometrial cancer patients. Gynecol. Oncol. 126, 176–179. 10.1016/j.ygyno.2012.04.013 22507532

[B25] Yoon G. S. Choi S. H. Woo S. I. Baek Y. S. Park S. D. Shin S. H. (2021). Neutrophil-to-Lymphocyte ratio at emergency room predicts mechanical complications of ST-segment elevation myocardial infarction. J. Korean Med. Sci. 36, e131. 10.3346/jkms.2021.36.e131 34002551 PMC8129614

[B26] ZengW.DengH.LuoY.ZhongS.HuangM.TomlinsonB. (2024). Advances in statin adverse reactions and the potential mechanisms: a systematic review. J. Adv. Res. 10.1016/j.jare.2024.12.020 39681285

